# Electrophysiological insights into deep brain stimulation of the network disorder dystonia

**DOI:** 10.1007/s00424-023-02845-5

**Published:** 2023-08-02

**Authors:** Denise Franz, Angelika Richter, Rüdiger Köhling

**Affiliations:** 1 Oscar Langendorff Institute of Physiology, University Medical Center Rostock, Rostock, Germany; 2 Institute of Pharmacology, Pharmacy and Toxicology, University of Leipzig, Leipzig, Germany

**Keywords:** Dystonia, Deep brain stimulation, Cortico-basal ganglia-thalamo-cortical network, Striatal synaptic plasticity, Dopaminergic dysfunction, Animal models of dystonia

## Abstract

Deep brain stimulation (DBS), a treatment for modulating the abnormal central neuronal circuitry, has become the standard of care nowadays and is sometimes the only option to reduce symptoms of movement disorders such as dystonia. However, on the one hand, there are still open questions regarding the pathomechanisms of dystonia and, on the other hand, the mechanisms of DBS on neuronal circuitry. That lack of knowledge limits the therapeutic effect and makes it hard to predict the outcome of DBS for individual dystonia patients. Finding electrophysiological biomarkers seems to be a promising option to enable adapted individualised DBS treatment. However, biomarker search studies cannot be conducted on patients on a large scale and experimental approaches with animal models of dystonia are needed. In this review, physiological findings of deep brain stimulation studies in humans and animal models of dystonia are summarised and the current pathophysiological concepts of dystonia are discussed.

## Introduction

For decades, clinicians and scientists have searched for a model of the pathophysiology of dystonia, the third most common movement disorder after Parkinson’s disease and essential tremor with a prevalence of 0.03 to 0.06% of the population [30, 33]. Dystonia is a heterogeneous hyperkinetic neurological movement disorder characterised by sustained or intermittent involuntary muscle contraction, which leads to abnormal and often repetitive movements or postures, or both [2].

In 2013, dystonia was reclassified into a bi-axial system, with Axis I referring to clinical characteristics, and Axis II to aetiology. This system is now widely used [2, 33]. If dystonia is the only motor dysfunction, it is classified as isolated dystonia (previously referred to as “pure”, “idiopathic”, or “primary”). Dystonia may also occur in combination with other movement disorders (also known as secondary dystonia in the past), such as myoclonus or parkinsonism [2]. In the following, the term “dystonia” only refers to isolated dystonia in this review. It is hard to clarify the pathophysiology of the broad spectrum of symptoms and multiple causes, including several genetic types of dystonia [106]. Even if dystonia affects several muscle groups, the origin of the pathomechanisms is the central nervous brain. Numerous studies emphasise the role of the cortico-basal ganglia-thalamo-cortical and cerebellar-thalamo-cortical networks in the pathophysiology of dystonia, whereby different forms of dystonia may have different origins. In multiple brain regions, such as the basal ganglia, thalamus, brainstem, or cerebellum, microstructural abnormalities have been reported for a few specific forms of dystonia [66]. Although lesions can cause dystonia, there is generally no evidence of anatomical abnormalities in isolated dystonia [9]. Instead, it is debated that genetic and functional network factors cause dystonia. Here, several brain regions may be differentially affected, and various forms of dystonia may include several responsible genes, which makes it hard to treat patients with dystonia optimally. Apart from dopa-responsive dystonia, there is no rational treatment option or a causal therapy (also due to the lack of a clear pathophysiological concept). Symptoms are usually treated by using physical (e.g. physiotherapy, transcranial magnetic stimulation), pharmacological (e.g. botulinum toxin injections, anticholinergic drugs), and surgical approaches in drug-refractory dystonia (e.g. deep brain stimulation, pallidotomy) [112]. Deep brain stimulation (DBS) appears to be the most promising procedure for treating [33]. Despite an increase in clinical trial datasets, it remains hard to predict the outcome of DBS for individual patients [121], which also depends on careful patient selection [22, 60]. To improve efficacy and reduce the risks of stimulation-induced side effects such as parkinsonism and dysarthria [82, 128], research and development are increasingly adopting the approach of adaptive DBS systems consisting of responsive, adaptive, and closed-loop control modes [62]. However, such adaptive DBS systems require electrophysiological biomarkers, e.g. local field potentials, to predict dystonic symptoms. These biomarkers can be used as a signal for punctually turning on the DBS within a closed-loop system to obviate dystonic movements.

This review summarises current pathophysiological concepts of isolated generalised dystonia. We are concentrating on recent electrophysiological findings helping to elucidate the pathomechanisms of generalised dystonia. Since it would go beyond the scope of the present review, here we do not dwell further on genetic factors and refer to the work of Christine Klein [57]. In addition, we discuss recent electrophysiological results from DBS studies in humans and animal models complemented by our hypotheses on the mechanism.

## Hypotheses for the pathophysiology of isolated dystonia

**Fig. 1:**
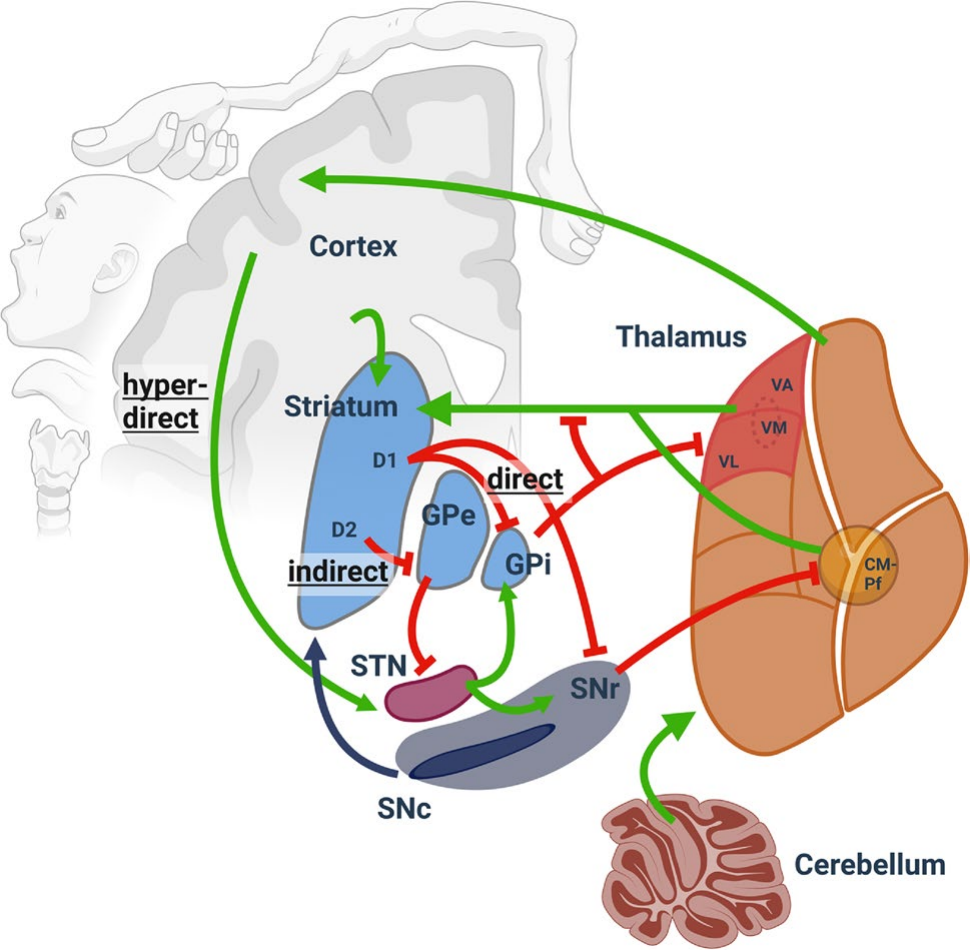
The cortico-basal ganglia-cerebellar-thalamo-cortical network. Movement formation is controlled by the motor circuitry, where the cortex, basal ganglia, cerebellum, and thalamus are interconnected and partly linked via collaterals, forming feedforward and feedback loops. Three pathways can be distinguished from each other: (1) the direct pathway, responsible for desired movements; (2) the indirect pathway is classically seen as a damping mechanism for undesired movements; and (3) the hyperdirect pathway, which bypasses the striatum and forms a monosynaptic connection from cortical areas to STN, mediating rapid movement inhibition. The green arrows represent glutamatergic inputs, the dark blue arrows represent dopaminergic inputs, and the red arrows represent inhibitory GABAergic inputs. The scaling of the nuclei is larger than the real anatomical sizes. Abbreviations: D1/D2, dopaminergic receptors class 1/2; STN, subthalamic nucleus; GPe/GPi, globus pallidus externus/internus; SNr/SNc, substantia nigra pars reticulata/compacta; VA, ventral anterior thalamic nucleus; VL, ventrolateral thalamic nucleus; VM, ventromedial thalamic nucleus; VA-VL-VM are part of the ventral motor thalamic nuclei (in red); CM-Pf, thalamic center median/parafascicular complex (figure created with BioRender.com)

Movement is controlled through the cortico-basal ganglia-thalamo-cortical loop of the central nervous system. Information from the cortex is directed to the striatum, a part of the basal ganglia, through glutamatergic afferents. The striatum modulates the information via cholinergic and GABAergic interneurons as well as dopaminergic and glutamatergic afferents from the midbrain and thalamus, respectively. Experimental studies established the first link between dystonia and a functional disturbance of the basal ganglia, especially in the cortico-striatal network [32, 95]. It was then thought that an imbalance between the direct and indirect pathways leads to excessive movement, but conclusive evidence for dystonia in humans is lacking [87]. As shown in Fig. 1, in the striatum, the cortical glutamatergic input is directly relayed to the globus pallidus internus (GPi), and the substantia nigra pars reticulata (SNr) (direct pathway). Moreover, cortical input is indirectly interconnected to the GPi via the globus pallidus externus (GPe) and the subthalamic nucleus (STN) (indirect pathway). For the sake of completeness, the hyperdirect pathway should also be mentioned here, which bypasses the striatum and forms a monosynaptic connection from cortical areas to STN, mediating rapid movement inhibition [20]. Basal ganglia output from the GPi/SNr is routed to ventral motor thalamic nuclei and forwarded to the frontal cortical areas. Moreover, the collaterals of the GPi/SNr projections run to the two primary components of the intralaminar thalamic nuclei, the center median (CM) and parafascicular (Pf) nuclei [125] (Fig. 1). CM and Pf neurons return projections to the striatal projection neurons (MSN; medium spiny neurons) and the aspiny interneurons. These interneurons may act as a feedback system that provides information on sensory events and are likely essential for arousal, attention, orientation, and action selection [64, 125]. In the classical model of basal ganglia function, the direct pathway is responsible for and facilitates desired movements, whereas the indirect pathway (although it can be co-activated for selected actions [21]) attenuates and inhibits unwanted movements [19, 39].

### Role of GABAergic inhibition

Regarding functional network factors, previous animal and human studies suggest reduced excitability of inhibitory connections at the cortical, brainstem, and spinal levels and a partial loss of inhibition [46]. The *loss of inhibitory tone within the striatum* is indeed one possible mechanism discussed for the pathophysiology of dystonia.

**Fig. 2:**
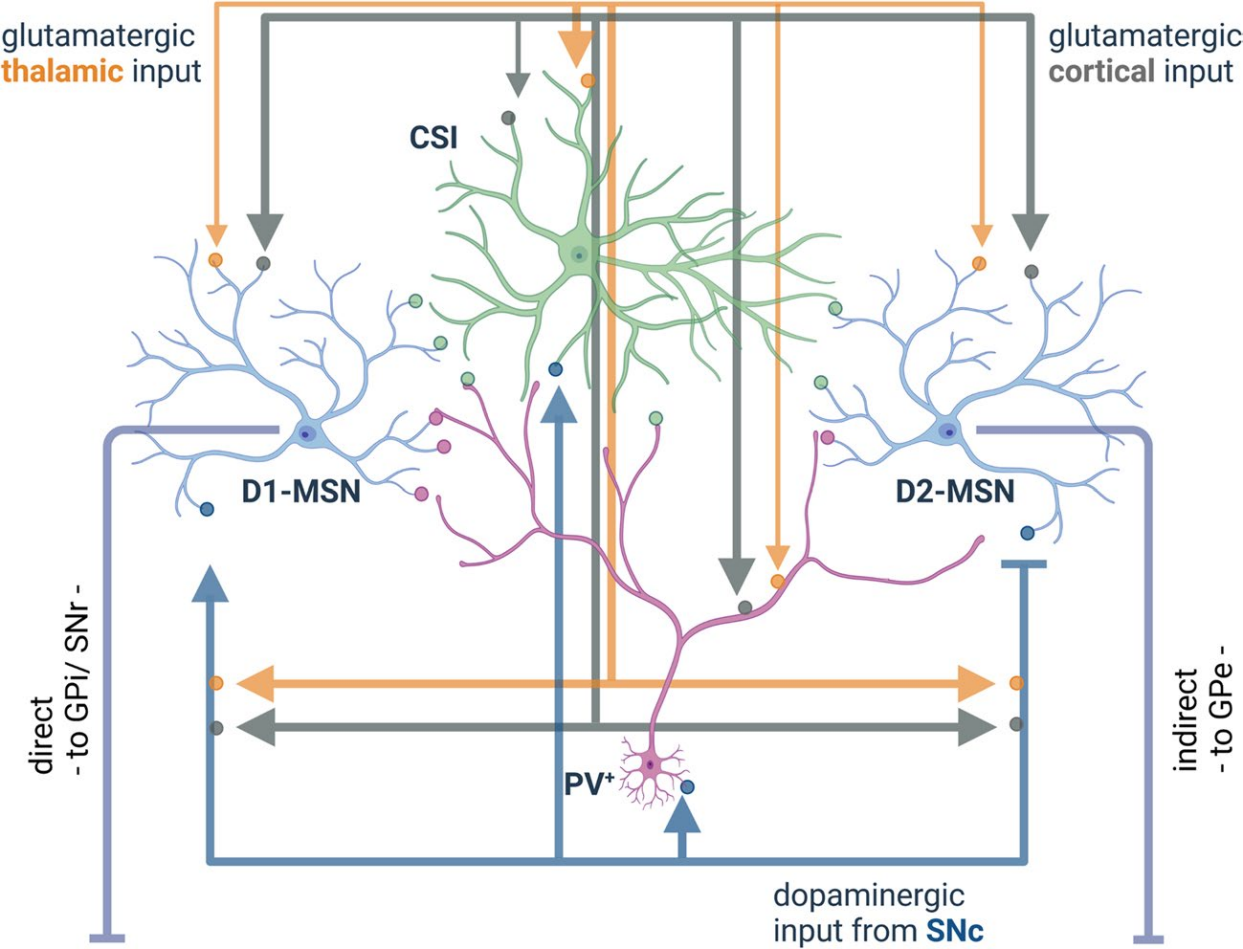
The intrastriatal network. The striatum plays an essential role within the movement loop and relays the glutamatergic cortical input. The striatal output to GPi (direct pathway) and the GPe (indirect pathway) is mediated through GABAergic projection neurons (medium spiny neurons, MSN), in which D1 or D2 receptors predominate, respectively. This striatal inhibitory output is modulated e.g. by GABAergic (PV^+^; parvalbumin-positive, purple) and cholinergic (CSI, green) interneurons as well as dopaminergic input (blue) from the substantia nigra pars compacta (SNc), glutamatergic thalamic, and cortical inputs. The small dots on the neurons and connections represent the appropriate dopaminergic, glutamatergic, and GABAergic synapses (figure created with BioRender.com)

The striatal GABAergic projection neurons, the MSN, represent the main proportion of striatal neurons (~95 %). The remaining ~5 % are interneurons, however, significantly impacting striatal output. Three-quarters of the interneurons comprise GABAergic interneurons, which can be divided into five classes: fast-spiking, 5HT3a-expressing, neuropeptide Y-expressing, calretinin-expressing, and tyrosine hydroxylase-expressing [111]. In the dt^sz^ mutant hamsters, an animal model of paroxysmal generalised dystonia, the maturation of fast-spiking striatal parvalbumin-positive GABAergic (PV^+^) interneurons is disturbed [13, 37]. Although the PV^+^ interneurons represent less than 5 % of total neurons in the striatum, the fast-spiking characteristic of these neurons (10–100 Hz) enables balancing MSN’s excitation coming from thousands of cortical glutamatergic excitatory inputs [39]. These fast-spiking interneurons balance the striatal activity and control the inhibitory tone of the striatum. PV^+^ interneurons are more responsive to excitatory cortical input than MSN, and due to their frequency-independent short-term synaptic plasticity, PV^+^ interneurons act as an attenuating filter for the MSN. Notably, the feedforward inhibition by PV^+^ interneurons of the MSN of the direct pathway seems to be stronger than that of the indirect pathway (Fig. 2) [39]. Therefore, a deficit of PV^+^ interneurons may have a more significant impact on the GABAergic MSN of the direct pathway and thus result in increased inhibition of GPi, which would consequently lead to less inhibition of the thalamus and could cause hyperkinetic movements. Single-unit recordings in the entopeduncular nucleus (EPN; homolog of the human GPi) of the dt^sz^ mutant hamster confirm this assumption and indicate a reduced mean discharge rate [36]. However, a decreased firing rate of the GPi cannot be considered the primary cause of dystonia due to the ameliorating effect after pallidotomy (which is typically restricted to the sensorimotor portion of GPi) [36]. Therefore, an abnormal pattern of GPi activity may be more relevant than an altered discharge rate [36].

Current theories stress the importance of *spatial and temporal coding of activity patterns* within GPi and SNr in the context of the overall network activity of the cortex, basal ganglia, thalamus, and cerebellum. Detections of local field potentials, which reflect the synchronised oscillatory synaptic activity surrounding a measuring electrode, demonstrated abnormal activity patterns with excessive pallidal low-frequency oscillation (delta and theta range; 4–12 Hz) in correlation to motor symptom severity in patients with cervical dystonia [79, 105]. Turning back to experimental data, in vivo extracellular single-unit recordings of the dt^sz^ mutant hamster with delayed maturation of striatal PV^+^ interneurons demonstrated a significant shift toward an irregular burst-like firing of EPN neurons in comparison to non-dystonic control animals [36]. Neumann and colleagues, in addition, showed reduced pallido-cerebellar connectivity, the synchrony of which was inversely correlated to the degree of symptom severity in dystonia patients [70, 79]. The cerebellum, on his part, appears indirectly connected to the striatum via the intralaminar thalamic nuclei, which modulate striatal dopamine release on synapses of dopaminergic midbrain neurons [113]. Excessive pallidal theta activity may thus change the spatial and temporal coding in the basal ganglia-thalamic-cerebellar loop in total and lead to abnormal striatal transmitter release and the generation of dystonic muscle contraction.

### Role of dopaminergic activity

Several dystonia forms have also been linked to *dopaminergic dysfunctions*. The neurotransmitter dopamine is released by afferents from the midbrain. Here, neurons from the ventral tegmental area (VTA) innervate the ventral striatum and neurons from the substantia nigra pars compacta (SNc) innervate the dorsal striatum. Dopamine affects the MSN directly via the D1-class or D2-class dopamine receptors (D1-DR or D2-DR, respectively) or indirectly via synaptically coupled neurons, e.g. GABAergic and cholinergic striatal interneurons (CSI). MSN directly projecting to the GPi/SNr (striatonigral) express high levels of D1-DR, while MSN of the indirect pathway (striatopallidal) strongly express D2-DR [110].

Dopamine concentrations and related metabolites are decreased in the dopa-responsive dystonia syndrome (Segawa syndrome), characterised by mutations in genes encoding enzymes involved in dopamine syntheses such as GCH1, TH, and SPR [9]. That dystonia form typically manifests as lower limb-onset dystonia and is characterised by a diurnal fluctuation of symptoms. Patients with dopa-responsive dystonia respond very well to treatment with the dopamine prodrug levodopa, even at low doses. In contrast, dopamine-depleting drugs sometimes improve other types of dystonia [112].

In DYT1 and DYT6 dystonia, the most common inherited forms of generalised dystonia, neuroimaging studies in patients and animal models revealed a significant reduction in the D2-DR availability and binding in the striatum [3, 15, 18]. In contrast to the D1-DR, primarily located on the postsynaptic site, the D2-DR can also be found at presynapses of glutamatergic and dopaminergic afferents and directly autoregulate the dopamine level in the striatum [23]. The D1-DR is coupled to G_olf_-protein, whose activation increases the intracellular cAMP level that leads to the activation of protein kinase A and affects various intracellular targets, e.g. voltage-dependent Na^+^ and Ca^+^ channels. The D2-DR is coupled to G_i/o_ protein, whose activation leads to adenylyl cyclase inhibition and phospholipase C stimulation. Overall, the activation of D1-DR facilitates the direct pathway by increasing the excitability of striatonigral MSN, while D2-DR activation restricts the indirect pathway by reducing the excitability of striatopallidal MSN [109]. Due to the heterogeneous distribution of receptors and synaptic inputs in the striatum, the overall effect of a dopaminergic dysfunction on the striatal output is hard to predict. Although reduced striatal D2-DR binding has been demonstrated in patients with DYT1 or DYT6 dystonia, animal models carrying the same DYT1 mutation and showing reduced striatal D2-DR binding are absent the dystonic motor symptoms [54]. However, in the symptomatic animal model of the dt^sz^ mutant hamster, which displays the type of paroxysmal generalised dystonia, autoradiographic analyses demonstrated decreased dopamine D1- and D2-DR in the dorsomedial striatum [80]. In another study that investigated the striatal extracellular levels of dopamine by microdialysis, dopamine release was significantly increased in correlation with a dystonic attack in the dt^sz^ mutant hamster [48]. Together with the findings in humans, these results suggest that overactivity of the dopaminergic system may be critically involved in the pathophysiology of dystonia. Besides the MSN, the D1- and D2-DR have also been identified in GABAergic interneurons and CSI. However, the interaction of dopamine and acetylcholine appears to play a crucial role in modulating striatal output [14, 32].

### Role of cholinergic activity

An alternative pathophysiological hypothesis of dystonia assumes *abnormal activity of cholinergic striatal interneurons* (CSI) related to acetylcholine-dopamine imbalances [24, 32, 47, 85, 100]. CSI are a subgroup of tonically active neurons (TAN) which can be identified due to the expression of choline acetyltransferase. CSI are morphologically characterised by a sizeable polygonal soma (average 20–50 μm), aspiny dendrites, and an axon that branches densely and abundantly. Despite a small fraction of approximately 2 % of the overall striatal neuronal population, CSI regulate the duration, strength, and spatial patterns of striatonigral and striatopallidal MSN [102]. Due to a persistent Na^+^ current (via Na_V_1.6), Ca^+^-activated K^+^- currents (via Ca_V_2.1, Ca_V_2.2, BK- and SK- channels), and a hyperpolarization-activated cation current I_f_, CSI show an autonomous activity of rhythmic single spiking (~5 Hz), even in the absence of synaptic inputs [12, 41, 78]. However, like TAN, the spontaneous firing pattern is synchronously paused, which is, on the one hand, mediated primarily by dopaminergic (acting via D2-DR) and partly by GABAergic afferents arising from SNc [27, 28, 96, 108]. On the other hand, the inhibition of CSI is mediated by intrastriatal GABAergic interneurons, for which tyrosine hydroxylase-expressing interneurons seem to be mainly responsible [28, 31]. This pause could be shown to respond to reward-related stimuli and is thought to act as a temporal window for the induction of synaptic plasticity in MSN [28, 41, 101]. Moreover, the synchronised pause exhibits variable patterns concerning the aforegoing bursts and the rebound spiking mediated by the CM-Pf complex [28, 96]. In the mouse model of DYT1 dystonia, an abnormal pause response in CSI was reported after repetitive thalamic stimulation [99]. It has been shown that the pause abnormality is caused by an altered activity of the D2-DR in CSI, which paradoxically excites the CSI rather than inhibiting it, as would be expected physiologically [32, 99]. A possible reason for the paradoxical effect of the D2-DR activity is an increase in the inhibitory coupling of D2-DR to the Ca_V_2.2 channels, which, in turn, reduces Ca^2+^-activated SK-currents in CSI [99, 100]. Consequently, the abnormal activity of CSI affected the cortico-striatal synaptic communication of the MSN at presynapses via M4 and at postsynapses via M1 receptors [99].

Acetylcholine modulates the activity of striatonigral MSN through muscarinic M1 and M4 receptors, while in striatopallidal MSN, the M1 receptor is the predominant one. M1 receptors are coupled to G_q/11_ and are found on the dendrites and spines. Activation of M1 receptors enhances NMDA-receptor mediated currents and persistent Na^+^ currents, modulates Ca^2+^-currents, reduces KCNQ and Kir2 currents, and in the end, promotes depolarisation of MSN [1, 32]. The M4 receptor activation (coupled to G_i/o_) leads to an inhibition of adenylate cyclase (AC) that mediates the inhibition of Ca_V_2.1 and Ca_V_2.1 [1]. The suppression of AC also opposes the D1-DR-induced cAMP/protein kinase A (PKA) signalling pathway in the striatonigral MSN, which induces long-term potentiation (LTP) at cortico-striatal synapses [1, 32, 78, 101]. It has been shown that M4 and D1 receptors interact asymmetrically in striatonigral MSN. That means that activation of the M4 receptor before activation of the D1 receptor leads to increased action potential frequency of striatonigral MSN. In contrast, prior activation of the D1 receptor causes a decrease in action potential frequency [51, 78].

Muscarinic acetylcholine receptors are widely expressed and identified on all striatal neurons, as autoreceptors on CSI, and on the axon terminals of most striatal afferents, except on dopaminergic midbrain neurons, where there is no anatomical evidence for the presence [5, 40, 113]. The expression of nicotinic acetylcholine receptors, with α7 and α4β2* as the most common subtypes in the striatum, is limited to the postsynapse of striatal interneurons and presynapse of striatal afferents [5]. The latter plays a crucial role in releasing dopamine from inactive midbrain afferents. With the help of fast-scan cyclic voltammetry, Threlfell and colleagues measured dopamine release extracellularly on presynapses of the non-active midbrain neurons due to endogenous striatal acetylcholine release via CSI [113].

Conversely to the modulation of acetylcholine on dopaminergic neurons, dopamine modulates the CSI through D2 and D5 receptors, at which D2 postsynaptic receptors have been identified as the key mediators whose activation leads to reduced release of acetylcholine [32]. As mentioned above, in DYT1 dystonia, D2-DR activation leads to a paradoxical excitation of the CSI [99]. Interestingly, imaging studies in human DYT1 dystonia patients indicated an age-dependent lower expression of vesicular acetylcholine transporters (VAChT) in the striatum and cerebellum [73]. VAChT is involved in the vesicular storage of acetylcholine in the presynapses of cholinergic neurons and is a potent indicator for acetylcholine release. A reduced VAChT expression and, therefore, a possible decrease in the extracellular acetylcholine levels within the striatum in young symptomatic humans seem to contrast with the hypothesis of hypercholinergic activity in the asymptomatic DYT1 mouse model. Mazere et al. discuss that contradiction as a consequence of synaptic physiology and disease duration [73]. Decreased VAChT expression was detected only in young human patients, where the putamen is involved in procedural learning of motor routines. In older patients with a lower learning demand, there is no aberrance in VAChT level against healthy controls. Age-dependent changes in the VAChT level were interpreted as a compensatory mechanism for excessive CSI activity induced by the abnormal D2-DR activity, which is again in line with the hypothesis from the DYT1 mouse model [73].

### Role of adenosine receptors

Endogenous adenosine should also be considered in the pathophysiology of dystonia [90]. High levels of adenosine receptors are expressed in the striatum. A2A adenosine receptors co-expressing with the D2-DR in striatopallidal MSN [17, 34, 117] are particularly interesting. A2A receptors are also present (although less frequently) in presynaptic excitatory and inhibitory terminals. Flagmeyer and colleagues proved a modulation of the cortico-striatal and thalamo-striatal glutamatergic neurotransmission in the presence of an A1 adenosine receptor agonist [34]. In a study by Tozzi and colleagues, the A2A and the D2-DR modulate the excitatory cortico-striatal transmission [117]. In the adult-onset dystonia DYT25, characterised by gene mutation encoding the stimulatory G-Protein G_αolf_ (GNAL), the cAMP pathway in the MSN is disordered. Since G_αolf_ is coupled to the A2A adenosine receptors of the striatonigral D2-DR MSN, the receptor becomes more and more attractive in the concept of the pathophysiology of dystonia [83].

### Role of glutamatergic thalamo-striatal and cortico-striatal projections

Several lines of evidence indicate abnormal cortico-striatal activity in dystonia [49, 72], but in addition to the cortical input, MSN receive quantitatively similar glutamatergic inputs through thalamo-striatal synapses [6, 26, 76] (Fig. 2). The thalamic glutamatergic innervation, mainly arising from the CM-Pf complex, has also been mentioned for CSI, GABAergic interneurons, and nigrostriatal terminals, and partially with contrastive effects regarding synaptic plasticity [6]. The initial glutamatergic release probability at the thalamo-striatal synapse formed on MSN appears higher than at the cortico-striatal synapse since the former expresses paired-pulse depression, in contrast to paired-pulse facilitation in the cortico-striatal synapse after repetitive stimulation [26, 40].

The thalamic inputs exert a significant excitatory influence on CSI, while PV^+^ interneurons and MSN were primarily excited through cortical inputs [6, 41]. Thalamo-striatal activity driven by salient stimuli is responsible for the burst and pause pattern in the CSI that is otherwise spontaneously active at low frequencies of approximately 5 Hz [27]. It has been demonstrated that the pause coincides with an increased discharge activity in dopaminergic midbrain neurons (SNc). Together with the findings from Reynold and colleagues [89], increased striatal dopamine level simultaneous to the pause in activity of CSI is speculated to underlie learning goal-directed behaviour very likely mediated by the basal ganglia-thalamo network.

### Role of striatal synaptic plasticity

It is commonly accepted that the strength of excitatory glutamatergic synapses on MSN is involved in motor skill acquisition. Dysregulation has been implicated in several movement disorders, including dystonia and Parkinson’s disease [69, 101].

The polarity of long-term plasticity (depression or potentiation) at excitatory synapses onto MSN appears to depend on the coincidence of phasic dopamine activity, pause in CSI activity, and depolarised MSN [89]. Long-term potentiation (LTP) on cortico-striatal synapses requires isochronic elevated dopamine release, paused CSI activity, and depolarised postsynaptic MSN. Otherwise, if one of them is missing, it results in long-term depression (LTD) or no effect [89]. Going into detail, in striatopallidal MSN, LTD requires the activation of G_i/o_ protein-coupled D2-DR, which decreases adenylate cyclase activity and silences A2A receptors that mediate LTP. Moreover, postsynaptic mobilisation of endocannabinoids through the metabotropic glutamate receptor mGluR5 and L-type calcium channels induced LTD in the indirect pathway MSN [69, 101]. Endocannabinoids retrogradely diffuse across the synaptic cleft and activate the cannabinoid receptor CB1 at the presynapse. Activation of CB1 leads to decreased release probability of the presynapse and results in LTD. In direct-pathway striatonigral MSN, the mechanism of LTD induction by presynaptic activation of CB1 receptors seems similar to the indirect-pathway striatopallidal MSN. However, striatopallidal MSN does not express A2A and D2-DR, and, in contrast to the striatopallidal MSN, activation of the D1-DR prevents the induction of LTD [69, 101]. The critical mediator for LTD induction in striatonigral is assumed to be the G_i/o_ protein-coupled M4 receptor, whose role has not been investigated so far [69, 101]. However, the M4 receptor may act similarly to the D2-DR in striatopallidal MSN by decreasing the AC and suppressing the activity of the D1-DR [101].

LTP, as opposed to LTD, is only a mechanism in the postsynapse depending on NMDA receptor activation [69]. One unwanted adverse reaction of L-DOPA treatment in early- and mid-stage PD patients is dyskinetic movements, which are assumed to be pathological LTP of cortico-striatal synapses due to abnormal increase of D1-DR signalling [101]. In the striatonigral MSN of the direct pathway, activated G_olf_-protein coupled D1-DR led to increased activity of PKA. That, in turn, raises the phosphorylation of the NMDA receptor subunit NR2B, which enhances the receptor currents and may lead to LTP induction [69, 101]. In the indirect pathway MSN, the mechanisms are supposed to be similar to the direct pathway MSN, while enhancement of PKA activity is mediated by the A2A receptor [69, 101].

The complex mechanisms of synaptic plasticity of MSN reflect the crucial role of granular time-scaled activation of dopamine, acetylcholine, and adenosine receptors. It is widely assumed that plasticity at cortico-striatal synapses underlying motor learning is a critical factor in the pathophysiology of dystonia [8, 49, 87, 114]. There is evidence from clinical and experimental studies for *abnormal synaptic plasticity* and a prevailing in LTP, which lead to abnormal sensorimotor integration and consolidation of motor engrams [87]. The delayed effect of deep brain stimulation (DBS), which, in contrast to PD, could take several days, weeks, or even months, pointed out the abnormal plasticity that may be reorganised by the stimulation [10, 44, 49, 63, 84, 94, 124].

In summary, the current hypotheses on the pathophysiology of dystonia consider the dysfunctional effect on parts of the entire motor circuitry. The nuclei of the motor loop are interconnected, partly also linked via collaterals, and form feedforward and feedback loops. For several years, dystonia has been increasingly understood as an extensive network dysfunction involving the basal ganglia and the thalamus, cerebellum, and sensorimotor cortices [9, 56, 66, 70]. To elucidate the pathophysiology of the different forms of dystonia, the entire cortico-basal ganglia-thalamo-cortical network should be considered. Furthermore, cerebellar involvement has to be considered due to evidence of pathological cerebellar output transmitted to basal ganglia via the thalamus [88]. All in all, dystonia appears to be a *network disorder*, and besides the specific nuclei, the oscillatory patterns across the nuclei must be analysed. Due to the ethical limitations in human patients, especially due to the impossibility of obtaining data mirroring the physiological situation in healthy persons, symptomatic animal models are required to study the hypothesis of the pathophysiology of dystonia. In particular, multi-unit recordings in the mentioned brain areas are needed to elucidate the internal network communication patterns.

## Deep brain stimulation in dystonia

Deep brain stimulation (DBS) is an essential electroceutical treatment option that allows for adjustable stimulation in neurological and psychiatric disorders correlated with dysfunctional neural circuitry [71]. It is widely used to treat movement disorders such as Parkinson’s disease (PD), tremors, and dystonia. After receiving FDA and Conformité Européene (CE) approvals, treatment with DBS became the standard of care in movement disorders [62]. Current DBS systems for clinical use consist of intracranial electrode(s), extension wire(s), and an implantable battery-operated pulse generator. Due to the attachment of proteins and cells directly to the DBS electrode surface and an accumulation of extracellular matrix proteins and glial cells, the impedance of the electrode-tissue interface varies over time [68]. For DBS with voltage pulses, the varied impedance caused instabilities in voltage magnitudes. Thus, the voltage induced in the brain tissue would deviate from the target voltage and require frequent programmed parameter adjustment. Current-controlled DBS, in contrast, minimises these voltage fluctuations generated by impedance changes [67]. Therefore, a current-controlled stimulation is preferred over voltage-controlled stimulation for an optimal DBS treatment.

Due to lower morbidity and reversibility, DBS has increasingly been preferred over thalamotomy or pallidotomy for dystonia [61, 97]. In 1977, Mundinger published the first data on short-term intermittent thalamic DBS (30 min several times per day for up to 8 months) in 7 patients with cervical dystonia [77]. Since then, many patients have been effectively treated with DBS. Fan and colleagues recently analysed the effectiveness of GPi-DBS and STN-DBS in different types of dystonia based on published literature with the help of the Meta-analysis of Observational Studies in Epidemiology [33]. They confirmed GPi- and STN-DBS to be safe methods and provided evidence for improvement of the quality of life in patients with dystonia. Symptoms of patients with focal dystonia generally improved better than patients with segmental dystonia, and patients with primary dystonia had a better response than those with secondary dystonia. All in all, the improvement of dystonia severity is reported by 50 to 60 % for patients with segmental or generalised or cervical dystonia treated with high-frequency pallidal (GPi) DBS [16, 25, 55, 65, 71, 120, 122, 123].

Electrophysiological data in patients with dystonia treated with DBS suggest modifications of the synaptic plasticity within the cortical motor circuit [114]. With electrophysiological studies from primates and patients, DBS can be described as a functional blockade of transmission of information, also known as the term “informational lesion”, that alters the activity of axons within the volume of tissue activated [42, 124]. However, DBS has to be more than a disconnection of the pathological area through the synaptic blockade. In imaging studies with positron emission tomography, DBS treatment enhanced the prefrontal or frontal cortico-basal ganglia-thalamocortical loop [124]. It is therefore assumed that DBS acts as a selective filter, which, on the one hand, functionally decouples the structure being stimulated. Moreover, on the other hand, it leads to activation of the structures within the volume of activated tissue, which results in a more regular firing pattern and changes in bursting activity already seen in local field recordings in patients treated with DBS [74, 79, 115, 124].

Given the diversity of dystonia in patients, such as type and age at the onset of dystonia, questions arise regarding optimal DBS targeting and adapted individualised stimulation settings. More data from specific dystonia subtypes are necessary, which are difficult to collect due to the heterogeneity of dystonia [106]. Matters are further complicated by the fact that target region choice and technical parameter settings are interdependent since different DBS targets appear to require other stimulation parameters. Thus, STN stimulation needs a significantly lower voltage and pulse width than GPi stimulation [10, 118], which seems to be due to the differences in nuclei volume [129]. Another aspect which makes it difficult to assess the effectiveness of DBS for dystonia is that improvement in tonic dystonic symptoms after pallidal stimulation may be delayed and take several days, weeks, or even months [10, 44, 63, 94, 124]. To bridge the lack of insufficient data from human patients with dystonia, translational research on animal models remains an essential approach for clarifying the pathophysiology of dystonia and providing insights into the mechanisms of DBS at the molecular and cellular levels. With this knowledge, we can develop effective treatment options for the different dystonia types. As mentioned below, pallidal theta oscillation is currently the most promising biomarker for individual-adapted DBS treatment of patients with cervical dystonia [79]. An abnormal activity pattern with excessive pallidal theta frequency oscillations can be used as a signal for turning on the DBS within the closed-loop system. However, most of the current DBS systems for chronic implantation in freely moving rodents are limited by the runtime or, especially for small rodents such as mice and hamsters, by volume restrictions [86], such that data on long-term stimulation remain rare. Concerning the variable clinical outcome of DBS, however, further studies are needed on the specific forms of dystonia in a search for significant biomarkers that allow for optimised and adapted DBS treatment. Such biomarker search studies cannot be performed on patients on a large scale and need to be flanked by experimental approaches. Thus, we need to uncover the pathophysiology of dystonia using animal models of dystonia [84].

## Electrophysiological data on DBS in dystonia models

During the last decades, the number of animal models for dystonia has risen due to the need to test hypotheses suggested by human studies, where it is often impossible to investigate the underlying physiologic, molecular, and cellular abnormalities. Rauschenberger et al. showed that results from animal studies are transferable to patients with dystonia, considering the movement-related oscillations as a robust parameter among the different species [88, 104]. The animal models for dystonia have been divided into etiologic and symptomatic (phenotypic) animal models. The etiologic model focuses on genetic predisposition and often lacking motor symptoms in the phenotype [92]. The symptomatic model represents clinical features consistent with human disorders and is suitable for identifying anatomical and physiological processes that elicit motor symptoms [126]. However, there are very few symptomatic animal models of dystonia. We refer to the reviews, which summarise current animal models for dystonia [54, 92, 126], and discuss the suitability of non-human primate and rodent dystonia models for DBS research [84].

**Fig. 3:**
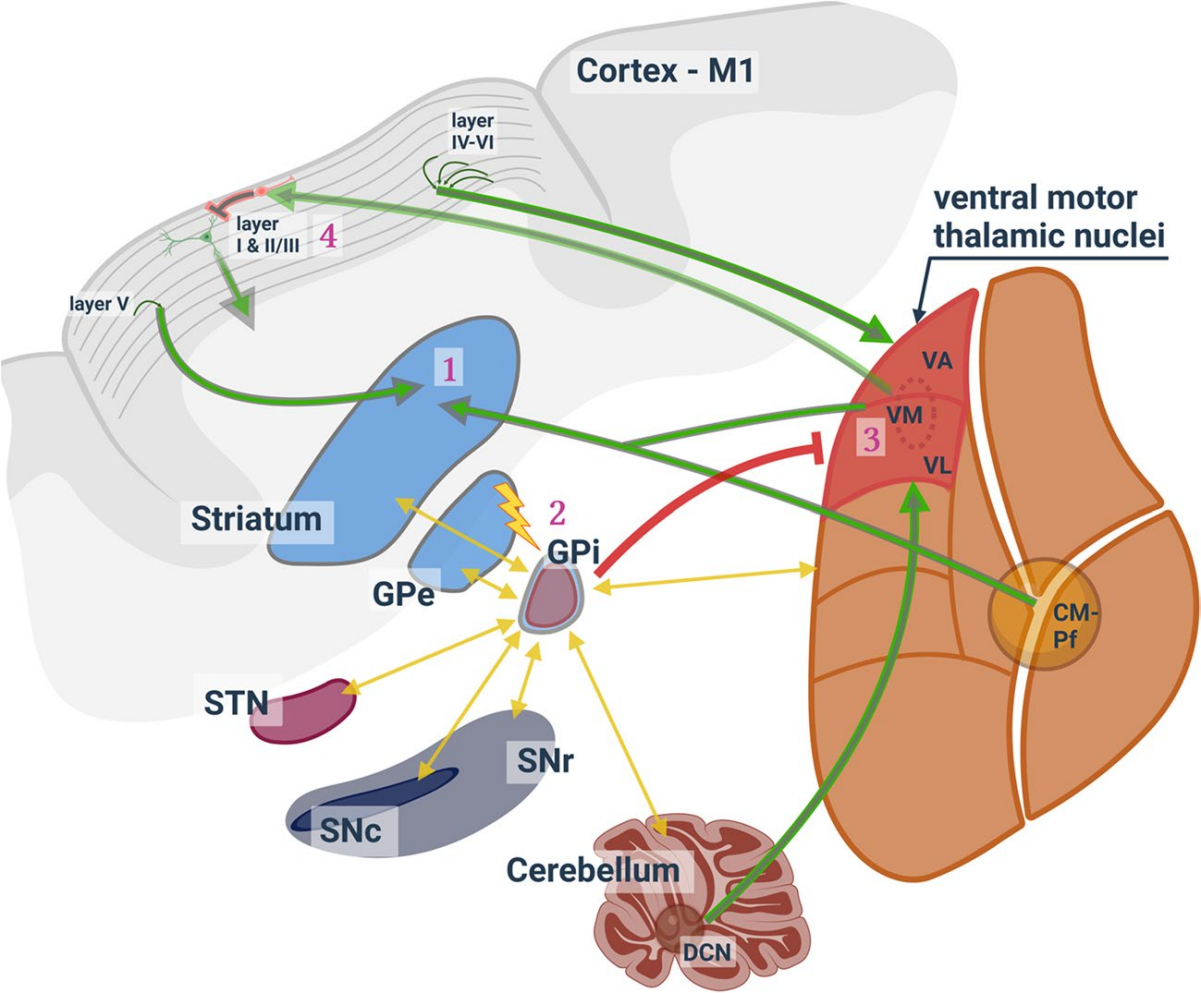
Effects of pallidal deep brain stimulation (DBS) in dystonia. Pallidal DBS is thought to be retrogradely and anterogradely relayed through reciprocal connections to several areas of the cortico-basal ganglia-thalamo-cortical loop (yellow arrows). Effects of pallidal DBS were already experimentally confirmed in dystonia animal models for the numbers 1–3: (1) Striatal medium spiny neurons showed reduced excitatory input provided through cortico-striatal and thalamo-striatal projections [49]. (2) DBS was also confirmed to reduce the pallidal theta power of the GPi [58]. (3) Inhibitory input to ventral motor thalamic nuclei (VA-VL-VM) neurons appears to be quantitatively unchanged. However, the excitatory input increased, which is thought to originate from afferents from the M1 (layers IV–VI) and the DCN. The hypothetical consequences of the increased excitatory input are summarised in the cartoon at site number (4): feedforward inhibition through inhibitory interneurons of layer I (which receive excitatory synaptic inputs from VM neurons) is strengthened. Inhibitory interneurons in layer I, in turn, dampen the activity of pyramidal neurons in layers II/III via their dendritic tufts, alleviating dystonic symptoms. Abbreviations: M1, primary motor cortex; STN, subthalamic nucleus; GPe/GPi, globus pallidus externus/internus; SNr/SNc, substantia nigra pars reticulata/compacta; VA, ventral anterior thalamic nucleus; VL, ventrolateral thalamic nucleus; VM, ventromedial thalamic nucleus; VA-VL-VM are part of the ventral motor thalamic nuclei (in red); CM-Pf, thalamic centre median/parafascicular complex; DCN, deep cerebellar nuclei (figure created with BioRender.com)

There are few studies of DBS on symptomatic animal models of dystonia. The dt^sz^ mutant hamster, a symptomatic model of paroxysmal dystonia, displays clinical symptoms of human dystonia patients in association with increased cortico-striatal excitability [7, 8, 59], and abnormal theta band oscillations [38]. Moreover, single-unit recordings indicated increased activity of the MSN and reduced activity of the EPN (entopeduncular nucleus, homologue of the human GPi), which likely leads to increased inhibition of the thalamus in the motor circuitry [11, 36]. Taking this model as a bona fide model of symptomatic dystonia, DBS was tested for its antidystonic efficacy, comparing STN-DBS (following the notion that STN can be an alternative target for DBS) and GPi-DBS in the dt^sz^ mutant hamster [10, 118]. Only GPi-DBS, but not STN-DBS, significantly reduced the severity score of dystonia within 3 h of stimulation in awake animals with no difference in the stimulation parameters (pulse frequency: 130 Hz, pulse width: 60 μs, current amplitude: 50 μA) [81]. Further, to test whether lower frequencies of GPi-DBS are as effective as 130 Hz, Paap and colleagues analysed the severity scores at 40 Hz and 15 Hz DBS with all other stimulation parameters remaining constant. Reducing the stimulation frequency incidentally would consume less power in a stimulation system, allowing for longer intervals between charging [86]. The experiments revealed that, at least for the dt^sz^ mutant hamster, 130 Hz is the most effective frequency for short-term DBS of 3 h. Taking the optimal GPi-DBS parameters as determined by Paap et al. (2021), we could show that the frequency of miniature excitatory postsynaptic currents (mEPSC) recorded in MSN of the dt^sz^ mutant hamster is significantly reduced [49]. Thus, compared to healthy, wild-type tissue, dt^sz^ mutant hamster neurons exhibited more mEPSC, which may reflect increased cortico-striatal excitability. In the same study, GPi-DBS was also conducted in normal, healthy, wild-type animals. In these, DBS had no significant effect on mEPSC, showing that the effect of DBS depends on the dysfunctional dystonic state of the network. Moreover, comparing mEPSC in healthy and dystonic tissues, DBS normalised mEPSC frequencies in dystonic tissue. Several explanations are possible for the reduced effect on MSN’s mEPSC: (1) DBS may retrogradely affect cortical or intrastriatal synaptic transmission via hyperdirect or direct pathway fibres. (2) Direct thalamic or indirect projections via the pallido-thalamo-cortical loop to the striatum are other plausible hypotheses [49]. (3) It is also conceivable that DBS affected the adenosine level, possibly by stimulating astrocytes [4], which influences excitatory cortico-striatal transmission [116, 117]. In summary, the reduced mEPSC frequency recorded in MSN after GPi-DBS in dt^sz^ mutant hamster can be interpreted as an adaptive process of the entire basal-ganglia network responding to DBS (Fig. 3).

As an example of a genetic dystonia model, the ΔETorA rat model genetically mirrors the most common inherited DYT1 dystonia due to an overexpression of the mutated human TOR1A gene. It lacks, however, a plain dystonic phenotype [43]. Such a dystonic phenotype emerges in this model only after inducing a severe peripheral nerve trauma by e.g. crushing the sciatic nerve, which results in dystonia-like movements of the hindlimbs (crushed ΔETorA rat). Though it cannot be excluded that sensory deafferentation or dysfunction also plays a role in the development of these dystonic-like movements, the crushed ΔETorA rat is used as a humanised genetic TOR1A dystonia model. Like the dt^sz^ mutant hamster, abnormal theta band oscillations can also be observed in the crushed ΔETorA rat [58]. Continuous GPi-DBS (pulse frequency: 130 Hz, pulse width: 200 μs, current amplitude individually adjusted to values 10 % below the occurrence of side effects) for 3 weeks significantly improved the dystonic-like movement severity score. Knorr et al. detected a slight reduction in theta power within the GPi that could be observed ~10 min after GPi-DBS was terminated [58].

The current findings are the beginning of understanding the efficacy of DBS on the network or, rather, on cellular mechanisms. However, previous experimental data are insufficient to elucidate the DBS mechanisms in dystonia. Most of the experimental data were determined with pallidal DBS. Data rarely exist for other stimulation targets in dystonia, such as STN or thalamus. In the following, we hypothesise the effects of pallidal DBS on the cortico-basal ganglia-thalamo-cortical loop in isolated dystonia, considering recent findings from animal experiments.

## Hypotheses on the mechanisms of DBS on dystonia

The electrical stimulation of brain tissue is a complex matter mainly due to the anisotropy of the tissue surrounding the electrode and the heterogeneity of the stimulated cells and structures [44]. The therapeutic mechanisms of DBS remain still unclear. Due to the similarity in therapeutic outcomes reached with DBS and thalamotomy or pallidotomy, it has been debated that high-frequency stimulation inactivates the structures being stimulated. This notion was also supported by findings showing decreased firing activity of the stimulated nucleus [10, 29, 74]. Cell membranes located directly at the cathode are generally depolarised, while membranes proximal to the electrode are hyperpolarised. Based on chronaxie measurements, it was suggested that GPi- and thalamic DBS primarily stimulates the afferents and efferents fibres rather than dendrites or cell bodies [53]. DBS will not affect the somatodendritic membrane if the pulse width remains below the membrane time constant (> 1 ms) [52]. Hence, the axons’ activity can be considered decoupled from the soma within a minimal distance of the stimulation electrode [44]. As a general finding, DBS induces more than just a local effect close to the stimulation site. It should be considered to evoke systemic effects as it produces action potentials that propagate orthodromically and antidromically, thus affecting the overall pattern of activity in the network through interconnected nuclei [4, 44, 75]. DBS may not correct the specific abnormalities that may cause dystonia (see above) but rather lead to a compensatory effect of the entire network that counteracts the pathomechanisms. Here, the continuous stimulation with a constant frequency may lead to alterations in the spatial and temporal coding of the activity patterns, probably affecting long-term synaptic transmission and plasticity, reflected in the delayed effect of DBS treatment in dystonia.

The reduction in excitatory input to striatal MSN, as recently reported [49], may lead to decreased striatal activity (Fig. 3). This, in turn, can lead to reduced inhibition of the GPi, which in turn would suggest increased inhibition of the thalamus, specifically the ventral motor thalamic nuclei, consisting of the ventrolateral (VL), ventral anterior (VA), and ventromedial (VM) nucleus. However, our first data from patch clamp recordings within the ventral motor nuclei of the thalamus of the dt^sz^ mutant hamster indicate that pallidal DBS does not affect the size of inhibitory postsynaptic currents [35]. Rather, there is a change in the inter-spike interval, which would be consistent with the reduced pallidal theta power in the study from the Ip group [58]. Alterations in the inter-spike interval would allow for synchronisation of the activity pattern with the stimulation frequency, rather than increasing the activity per se. In addition to changes in frequency of IPSC, EPSC in VL-VA-VM neurons in the DBS-treated group tended to be increased, again showing that DBS has network-wide effects [35]. Regarding the origin of the excitatory input to the ventral motor thalamic nuclei, the deep cortical layers (layer IV to layer VI) and the cerebellum are possible origins. What would be the effect of such increased excitation in thalamic neurons? Again, we can merely hypothesise that such increased excitatory input would strengthen feedforward inhibition through inhibitory interneurons of layer I, which receive excitatory synaptic inputs from VM neurons. The inhibitory interneurons in layer I inhibit pyramidal neurons in layers II/III over dendritic tufts [104], which could eventually alleviate dystonic symptoms. Alternatively, the increased excitatory input on the neurons of the ventral motor thalamic nuclei after long-term pallidal DBS may also result from strengthening the reciprocal positive feedback loop from the motor cortex (Fig. 3). On the whole, the impact of pallidal DBS can be retrogradely and anterogradely relayed through reciprocal and non-reciprocal connections to all areas of the cortico-basal ganglia-thalamo-cortical loop [45, 127] (Fig. 3).

Improvement of the dystonic symptoms can also be observed after thalamic DBS. In a study with a rat model of Parkinson’s disease, after 5-min electrical stimulation of the VA and VL, the number of induced action potentials in proximal neurons increased in vitro whole-cell patch clamp recordings [119]. VL neurons transitioned from bursting to non-bursting action potentials, while VA neurons were excited with little change in spiking phenotype. The authors speculated that thalamic DBS leads to increased excitation of the VA and VL neurons [119], which would be a similar effect as expected with pallidal DBS. The STN clinically is thought to be another promising stimulation target to improve dystonic symptoms [118]. However, at least experimentally, compared to GPi-DBS, short-term (3 h) STN-DBS did not alleviate dystonic symptoms in the dt^sz^ mutant hamster [81]. This discrepancy may be due to differences in focal dystonia in patients with effective STN DBS vs. generalised dystonia in the hamster model, where STN-DBS is ineffective. In the cited publication, it was reasoned that STN-DBS is influencing SNr activity and, hence, GABAergic projections to the CM-Pf complex, more than GPi-DBS (Fig. 1). The somatotopic organisation of STN and CM-Pf was thus thought to allow for the stimulation of pathways for specific muscle groups, given the correct localisation of the stimulation electrodes. For generalised dystonia, such specific stimulation was speculated to be insufficient.

Overall, it seems certain that one cannot expect an alteration exclusively in a single projection within the cortico-basal ganglia-thalamo-cortical loop or one specific ion channel through DBS; rather DBS alters the entire network, likely resulting in an adaption of the activities of the specific nuclei within the movement loop to the electrical stimulation. Although DBS is thus causing a network effect, that obviously does not exclude effects on specific ion channel compositions and transmitter release resulting from altered neuronal activity and plasticity.

The choice of the ideal stimulation target, in turn, likely depends on the degree of abnormal network activity. Pallidal DBS seems more effective in generalised dystonia due to a more extensive effect within the entire cortico-basal ganglia-thalamo-cortical loop. In contrast, STN-DBS appears to be better suited for focal dystonia, where the cortico-basal ganglia-thalamo-cortical loop likely is associated with abnormal activities only in specific pathways. The next step in dystonia research is to identify the optimal parameters for balancing the alterations of DBS effects and pathomechanisms leading to standard physiological motor control.

## Outlook

More knowledge on the pathophysiology of specific dystonia subtypes is necessary to find biomarkers for developing adaptive closed-looped DBS systems. With the help of translational research on animal models, we can overcome the lack of insufficient data from human patients with dystonia. We may obtain a detailed view of the pathophysiological and therapeutic (by DBS) mechanisms. Several in vivo, in vitro, and in silico methods help clarify the processes in the motor circuitry. Using microelectrode arrays (MEAs), simultaneous high-density recordings of the different nuclei in the cortico-basal ganglia-thalamo-cortical and cerebellar-thalamo-cortical network facilitate the investigation of network communication. Moreover, the MEA system enables defined local stimulation of specific parts in the network and the recording of spatio-temporal patterns in parallel that may be altered in dystonic tissue in our expectations. The MEA system also opens a new window for analysing synaptic plasticity (LTP and LTD) with a more significant number of recordings in parallel across a large area of the motor circuitry.

Translational research in animal models enables better access to tissue, which can be quantified for changes in cellular, molecular and transmitter levels affected by DBS. With the liquid chromatography-tandem mass spectrometry, e.g. microdialysates from different time points of the DBS were analysed to quantify the transmitter level [50]. Regional metabolic changes after DBS, already described for dystonia [80], are characterised by the positron emission tomography with the radiopharmaceutical glucose analogue (18)F-fluorodeoxyglucose. The investigation of specific local abnormalities, like the hypothesised striatal abnormal cholinergic tone, requires the individual pharmacological manipulation of these neurons for optogenetic experiments. In previous studies, the optogenetic activation of CSI [91] and striatopallidal MSN [98] in the DYT1 mouse model supports the detailed investigation of the already mentioned hypothesis of abnormal activity of CSI and imbalance between the direct and indirect pathway, respectively. The precise spatial and temporal selectivity of optogenetics that allows for stimulation of either afferent fibres or cell bodies makes it a powerful tool for studying the mechanisms of DBS [93].

For understanding and clarifying the complexity of the movement circuitry in detail, in silico modelling seems to be an additional option besides the experiments [103, 107]. The effects of variable DBS parameters can be modelled and tested in animal experiments in case of promising results.

## Data Availability

Not applicable.
